# Decoding and Systematization of Medical Imaging Features of Multiple Human Malignancies

**DOI:** 10.1148/rycan.2020190079

**Published:** 2020-09-11

**Authors:** Lu Wang, Zhaoyu Liu, Jiayi Xie, Yuheng Chen, Xiaoqi Zhao, Zifan You, Mingshu Yang, Wei Qian, Jie Tian, Kristen Yeom, Jiangdian Song

**Affiliations:** From the School of Medical Informatics, China Medical University, Shenyang, Liaoning, China (L.W., M.Y., J.S.); Department of Radiology, Shenjing Hospital of China Medical University, Shenyang, Liaoning, China (Z.L.); Department of Radiology, China Medical University, Shenyang, Liaoning, China (J.X., Y.C., X.Z., Z.Y.); Department of Electric and Computer Engineering, University of Texas–El Paso, El Paso, Tex (W.Q.); CAS Key Laboratory of Molecular Imaging, Institute of Automation, Chinese Academy of Sciences, Beijing, China (J.T.); and Department of Radiology, Stanford University School of Medicine, 1201 Welch Rd Lucas Center PS055, Palo Alto, CA 94305 (K.Y., J.S.).

## Abstract

**Purpose:**

To summarize the data of previously reported medical imaging features on human malignancies to provide a scientific basis for more credible imaging feature selection for future studies.

**Materials and Methods:**

A search was performed in PubMed from database inception through March 23, 2018, for studies clearly stating the decoding of medical imaging features for malignancy-related objectives and/or hypotheses. The Newcastle-Ottawa scale was used for quality assessment of the included studies. Unsupervised hierarchical clustering was performed on the manually extracted features from each included study to identify the application rules of medical imaging features across human malignancies. CT images of 1000 retrospective patients with non–small cell lung cancer were used to reveal a pattern for the value distribution of complex texture features.

**Results:**

A total of 5026 imaging features of malignancies affecting 20 parts of the human body from 930 original articles were collated and assessed in this study. A meta-feature construct was proposed to facilitate the investigation of details of any high-dimensional complex imaging features of malignancy. A correlation atlas was constructed to clarify the general rules of applying medical imaging features to the analysis of human malignancy. Assessment of this data revealed a pattern of value distributions of the most commonly reported texture features across human malignancies. Furthermore, the significant expression of the gene mutational signature 1B across human cancer was highly consistent with the presence of the run length imaging feature across different human malignancy types.

**Conclusion:**

The results of this study may facilitate more credible imaging feature selection in all oncology tasks across a wide spectrum of human malignancies and help to reduce bias and redundancies in future medical imaging studies.

**Keywords:** Computer Aided Diagnosis (CAD), Computer Applications-General (Informatics), Evidence Based Medicine, Informatics, Research Design, Statistics, Technology Assessment

*Supplemental material is available for this article.*

Published under a CC BY 4.0 license.

**Summary**

Systematic review of 930 studies about clinical medical imaging features of malignancies across 20 different anatomic regions led to the identification of the most prominent texture features that could be selected for future characterization in clinical oncology tasks related to image acquisition, preprocessing, detection, characterization, monitoring, and reporting.

**Key Points**

■ Systematization of medical imaging features in human malignancies may reduce the vibration of effects and redundancies in future medical imaging studies.
■ Application rules of medical imaging features in oncology tasks across human malignancies and the difference in visual characteristics of the malignancy images with different texture feature values were identified (*P* < .0001 in different feature value groups).
■ Connections between medical imaging features across human malignancies and the signatures of mutational processes across human cancer were identified (Kolmogorov-Smirnov test, *P* > .5).

## Introduction

Medical imaging is a critically important tool for assisting with the detection and treatment of human diseases, particularly malignancies (1). It is well established that subtle intratumoral heterogeneity information could be comprehensively extracted by decoding medical imaging features. In the past few decades, thousands of quantitatively and qualitatively engineered features extracted from both radiologic images and histopathologic images have been identified (2–5). With continuous advances in the technology of medical imaging feature analysis, previous studies have confirmed that high-throughput imaging heterogeneity descriptors are critical to revealing the occurrence, progression, and treatment response of human malignancies (6–9).

Despite the rapid developments in the field of medical imaging feature analysis, the general rules for the utilization of decoded medical imaging features to assist in various clinical tasks (characterization, detection, monitoring, acquisition, preprocessing, and reporting) related to different human malignancies remain unclear (10–12). Current approaches to assess medical imaging features have generally been based on massive feature extraction, after which machine learning methods are selected for feature screening to identify potential imaging biomarkers. Vibration of effects, which are the result of the preference and subjective experience of the investigators in this process, as well as adjustment of parameters of the machine learning methods, make the feature-screening process unreliable and difficult to reproduce (7,13). Moreover, because most studies do not use the same criteria to standardize feature extraction, an understanding of imaging features that can potentially influence tasks remains limited; redundant features are inevitably used (14–17). The redundancy of features and the inconsistent criteria used for feature extraction inevitably hamper the reproducibility and weaken the interpretation of research results (18,19). Therefore, there is a need to appropriately systematize the reported imaging features to provide a basis for a more robust feature selection in this field to address these challenges.

Standardized calculations and definitions of medical imaging features have been proposed by the Image Biomarker Standardization Initiative (IBSI) (20), which have boosted the standard imaging feature-set construction. Furthermore, RadLex, a new radiology lexicon resource with more than 40 000 standardized terms covering all aspects of radiology, has significantly advanced communication in radiology (21–23). In addition, other studies have also made recommendations for reducing imaging feature redundancy (13,17). However, because of a lack of understanding known imaging features associated with human malignancies, the interpretation of tumor heterogeneity reflected in medical imaging features remains controversial to this date (24). Thus, systematizing existing data of imaging features in human malignancies is warranted to provide a scientific basis for improving the reproducibility and clinical applicability of experimental results.

The aim of this study was to summarize previously reported, statistically significant medical imaging features of human malignancies to provide a scientific basis for more credible imaging feature selection in clinical tasks pertaining to the malignancies. Studies that used medical images to extract quantitative or qualitative imaging features to aid in malignancy-related clinical practice were comprehensively reviewed in this study to construct an atlas of correlation between human malignancies and medical imaging features.

## Materials and Methods

This study consisted of a retrospective literature review, as well as a retrospective assessment of non–small cell lung cancer (NSCLC) images for analysis of different texture features. Institutional review board approval was obtained for the included patients with NSCLC for imaging feature presentation. We declare that the authors have been in full control of all data and information submitted for publication.

### Literature Retrieval and Quality Assessment

We searched PubMed from database inception through March 23, 2018, for studies clearly stating the decoding of medical imaging features for malignancy-related objectives or hypotheses. The [Title/Abstract] field was used in the search strategy to find studies in these general research areas. We used the following search terms to obtain the studies for our analysis: “((image[Title/Abstract] AND cancer[Title/Abstract]) AND (feature[Title/Abstract] OR texture[Title/Abstract])).” All the retrieved full-text articles were downloaded and read by our investigators for information extraction. Then, the Newcastle-Ottawa scale (25), a critical appraisal tool for studies in systematic review, was used to assess the quality of all the articles we included in this review. See Appendix E1 (supplement) for details on article inclusion and exclusion criteria.

### Workflow of Manual Information Extraction

Ten study investigators, including five trainees in radiology (J.X., Y.C., Z.Y., X.Z., and L.W.), initially reviewed and extracted the articles independently, before September 2018; and four other radiologists and Z.L. with 6, 10, 15, 15, and 30 years of experience in radiology, respectively, performed double-blind in-house checking of the results in October 2018. All disagreements were finally blindly reviewed again by the five trainees in January 2019 to ensure accuracy of data abstraction. All the trainees had been trained in medical imaging feature extraction and had also finished a course in science literature reading. The procedure of extraction of the specified information was supervised by an expert (J.S.) with more than 7 years of experience in medical imaging feature analysis. To ensure the accuracy of data extraction, we randomly selected 30 original articles, and the information of all the articles was extracted by our investigators at the same time (blinded to the investigators).

### Categories of Extraction Criteria

To analyze the obtained articles, the following information was manually abstracted from each full-text article: *(a)* type of malignancy; *(b)* number of enrolled patients or images (if the authors did not provide the details of human patients); *(c)* radiology subspecialty and technique used; *(d)* clinical radiology tasks; and *(e)* name, definition, or description of the statistically significant medical imaging features reported in the study. For each study related to malignancy, we categorized each article by one of the following processes (designated 1 through 6): (1) characterization, (2) detection, (3) monitoring, (4) acquisition, (5) preprocessing, and (6) reporting. All names of imaging features reported to be statistically significantly correlated with the study purpose were manually identified and recorded by our investigators. For studies that only reported the final models for the prespecified tasks, without a description of the imaging features in the final model or articles that did not provide the details of the significant features, we extracted all the correlated features mentioned in the original text. All the information was manually identified and then stored in an Excel (Microsoft) spreadsheet.

### Meta-Feature Construction

The first challenge of medical imaging feature systematization consisted of identifying the names used for features. Although the definition of imaging features has been provided by the IBSI (20), in actual studies, the naming of features by different researchers is inconsistent (16). Examples of naming inconsistencies can be found within Appendix E2 (supplement).

On the basis of the complete list of all statistically significant imaging features that were manually extracted, we extracted each description that appeared in the list as a meta-feature. On the basis of statistics of these meta-features, it was then possible to clarify the details of utilization of each imaging feature in human malignancy-related tasks by recombining the meta-features. Details of the meta-feature construction can be found within Appendix E3 (supplement). The list of meta-features derived in this way are presented in Table E1 (supplement).

Previous studies indicate that imaging features are highly related to imaging methods. Therefore, our study provided descriptive statistics for the reported meta-features, based on the radiologic images (CT, mammography, MRI, PET, and US), as well as histopathologic images. In addition, for different clinical tasks (characterization, detection, monitoring, acquisition, preprocessing, and reporting), the highlighted imaging features differed. The details of the application of imaging meta-features across human cancers for each of the clinical tasks were also assessed.

Finally, to make it easier for readers to trace the information about original features from the meta-features, we provided statistical data on both the original features and the meta-features. By recombining the corresponding meta-features to a specific imaging feature, the utilization details of each original imaging feature across various parts of cancer and imaging modalities could be obtained.

Unsupervised hierarchical clustering was conducted by the pheatmap package in R (R Foundation, Vienna, Austria) to identify the distribution rules of imaging features across human malignancies. To clarify this discovery, we developed a visualized network topology diagram and translated the findings to an online tool to display our findings (*http://www.ciitool.com/#/mifa-correlation*).

### Study Design for Clinical Visualization of Significant Texture Features

A comprehensive PubMed search on lung nodule classification resulted in more than 1400 imaging features that have previously been reported to be statistically significant related to this single task, thus we aimed to determine the important texture features for NSCLC as a case example. We elucidated the pattern of distribution of the values of the most important medical imaging texture features (ie, those with highly reported frequencies across cancer).

Pretherapy contrast material–enhanced CT images of 1000 patients with early-stage NSCLC were collected in this study. Demographics of the patients are found in Table E18 (supplement). First, all tumor regions of interest (ROIs) on the images were manually segmented by two radiologists with more than 10 years of experience in chest CT interpretation. Then, texture feature extraction was performed on all the ROIs by using the standard calculation formula. Next, all patients were then classified into two subgroups by the median of the entropy value of the tumor ROIs. Finally, the feature values of entropy and other texture features were analyzed between the two subgroups.

In addition, 500 random images were generated by computer in this study, and the same texture feature extraction algorithm was used on the random images to further illustrate the visual differences between different texture features. On the basis of the features extracted from all the images, *F* test was used to evaluate the difference of features between each of the three image groups. The averaged fast Fourier transform (FFT) image of each group was then calculated by averaging the Fourier transforms of all images in the group. The averaged FFT (26) images were finally used as an intuitive representation of images with significantly different texture feature values.

### Association between Mutation Signatures and Imaging Features

An ad hoc exploration was performed to identify the potential connection between medical imaging features across human cancers and the signatures of mutational processes across human cancers. On the basis of the statistics of the expression of the medical imaging features across human cancers, we compared the presence of mutational signatures across human cancer types and the presence of medical imaging features across human cancer types. Specifically, we explored the potential association between mutations that were only significantly expressed in specific human cancers with the imaging features that were prominently presented in those cancer types.

The presence of each of the original imaging features across human cancers is shown in Table E2 (supplement) in this study. For each imaging feature, we defined the presence of the feature in a given cancer only if it was reported more than once for that cancer. The expression of mutation signatures in each human cancer was available in the work of Alexandrov et al (27) and Hoadley et al (28). To clarify this experiment, we have attached the results of the expression of the gene mutation signatures in human cancer published in those two previous studies in Figure E5 (supplement).

### Statistical Analysis

The intraclass correlation coefficient (ICC) was used as the consistency index (29) to analyze the consistency of information extraction from the 30 reports between different observers. We converted the qualitative data items to numbers; for the identical data items achieved between the observers, they are represented by the same number, and for the data items with difference, they are represented by different numbers. Then, the ICC was used to test the interobserver reliability of the two columns. We first calculated the ICC for each variable, and then the average ICC was used as the consistency assessment of data extraction between different observers. The Kolmogorov-Smirnov test was used to evaluate the distribution consistency of mutations that were only significantly expressed in specific human cancers and imaging features that were prominently presented in those cancer types. Pearson correlation coefficient was used to describe the similarity of the meta-feature data for each two different types of cancer. All statistical analyses were performed in R software (version 3.4.3). Texture feature selection was performed in MATLAB (version 2015b). The ICC was used to estimate the interobserver variability of the segmentation from the two radiologists. *P* < .05 was considered to be statistically significant.

## Results

### Overview of Included Studies and Malignancies

A total of 1105 studies were collected according to our search strategy. A total of 175 studies were excluded for the following reasons: *(a)* inappropriate study type (79 studies), *(b)* did not contain a record of descriptive medical images features (67 studies), and *(c)* the full text could not be obtained via the Internet (29 studies). After exclusion, 930 studies with approximately 370 000 research participants were included in the analysis (the flow diagram for the included studies is showed in Fig E1 [supplement]). Thirty-four types of malignancies, affecting 20 parts of the human body, were covered within the extracted studies (Appendix E1 [supplement]). Results based on the Newcastle-Ottawa scale indicated that 590 of the 930 studies (63%) were scored less than or equal to 3, 331 studies (36%) were scored between 4 and 6, and only nine studies (1%) were scored between 7 and 9.

**Figure 1:**
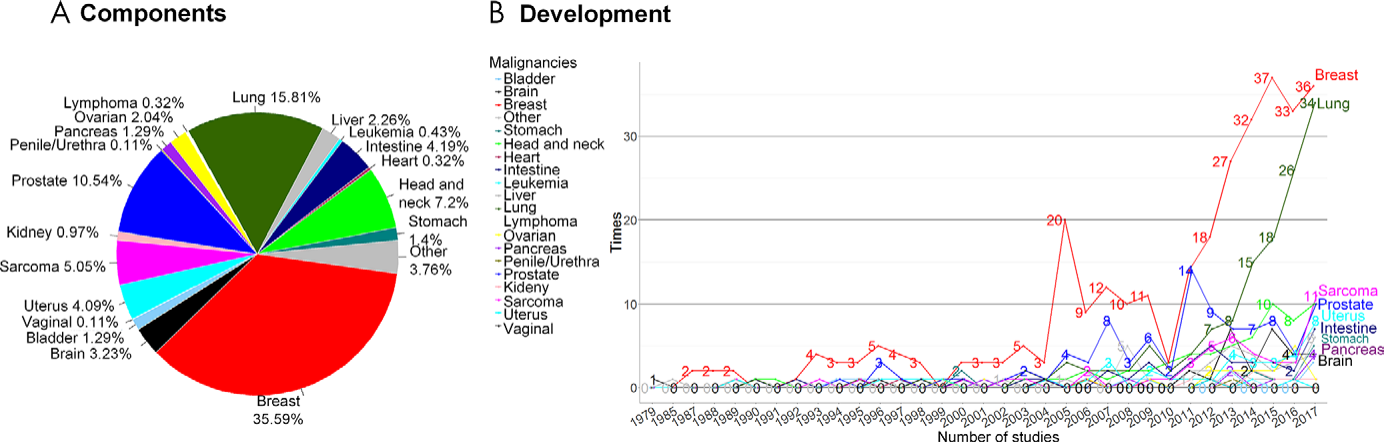
*A,* Distribution of studies of medical imaging feature analysis in human malignancies, and, *B,* development in the number of studies on different malignancy locations over time.

We found that the number of studies in the field of imaging malignancies significantly increased after 2004 (*P* < .0001, Fig 1). Most extracted studies were about breast (329 studies), lung (145 studies), and prostate (98 studies) cancer. From the 1980s to 2004, histopathologic images were the most-used method for malignancy assessment (59%, 64 of 108 reports). With the inception of other imaging technologies, studies based on MRI, CT, mammography, and US imaging were increasingly used in the following decade. After 2014, feature analysis based on CT images was predominant compared with the other three major imaging modalities.

### Overview of Extracted Medical Imaging Features

We found that 5026 clearly defined medical imaging features (from all 930 studies) were reported to be significantly correlated with specific clinical tasks related to human malignancy in the included studies (Table E2 [supplement]). To resolve the inconsistent naming of medical imaging features, a feature mapping set, including 450 meta-feature descriptors, was constructed according to the meta-feature construction method described in the methods section, based on the review of all 5026 original features. Details of the meta-features and corresponding original features are presented in Tables E2 and E3 (supplement). Reliable interobserver consistency of the data extracted by investigators in the current study was achieved (average ICC = 0.955).

**Figure 2:**
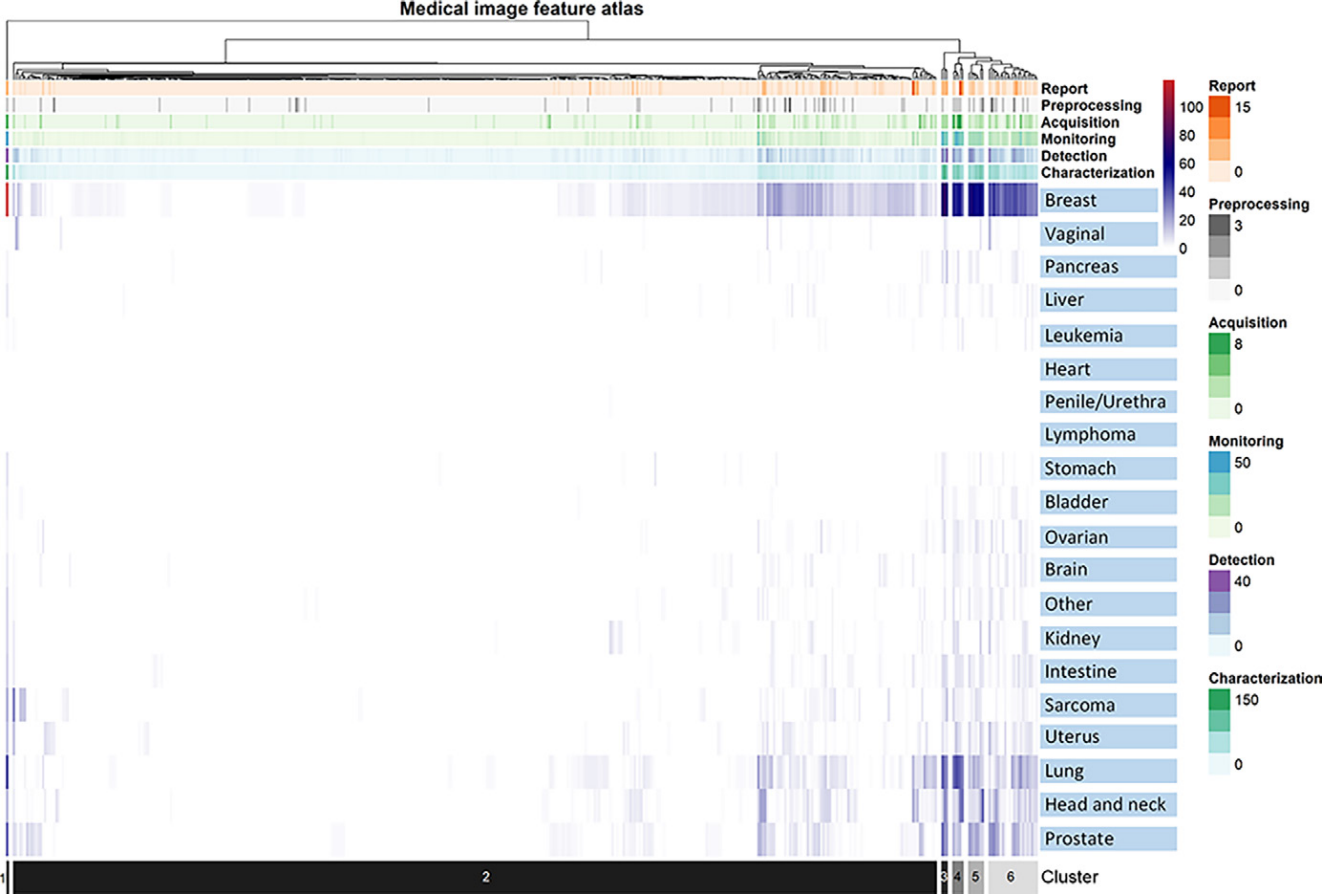
Unsupervised hierarchical clustering of medical imaging meta-features and cancer locations. The correlation between imaging meta-features and human malignancies is represented as a color gradient from low (white) to high (red). White indicates that there are no cited studies for the imaging feature and the malignancy type. Significant associations between the clinical oncology tasks and the distribution pattern of the meta-features: characterization (Pearson product-moment correlation, *r* = 0.834, *P* < .001), detection (*r* = 0.730, *P* < .001), monitoring (*r* = 0.845, *P* < .001), acquisition (*r* = 0.670, *P* < .001), preprocessing (*r* = 0.280, *P* < .001), and report (*r* = 0.490, *P* < .001).

**Figure 3:**
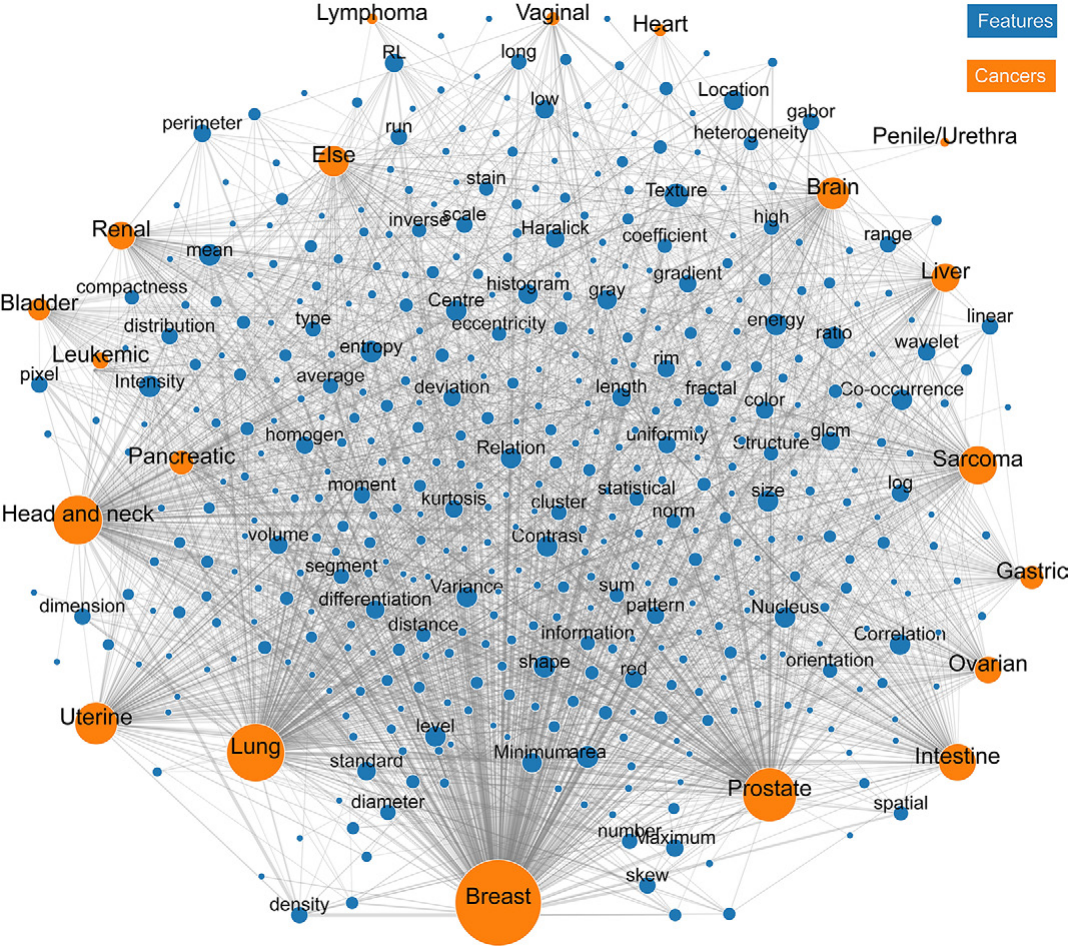
The visualized network topology diagram of the correlation atlas (see *http://www.ciitool.com/#/mifa*).

Figure 2 represents the results of unsupervised clustering of all pairs of entries for the correlation between human malignancies and image meta-features, the clustering of image meta-features across human malignancy types. Statistics on the frequency of meta-features reported in the six oncology tasks across human malignancies is presented in Table 1, and the characteristics of each cluster are presented in Table 2. The 20 anatomic regions and the associated corresponding meta-features associated are presented as a heatmap in which the correlation between them ranged from poor to strong. Moreover, the statistical analysis of meta-features applied to each clinical task was also represented by the heatmap in Figure 2. In addition, an online computing platform for visualization of the network topology diagram of meta-feature and human malignancies is shown in Figure 3 (Appendix E4 [supplement]).

**Table 1:**
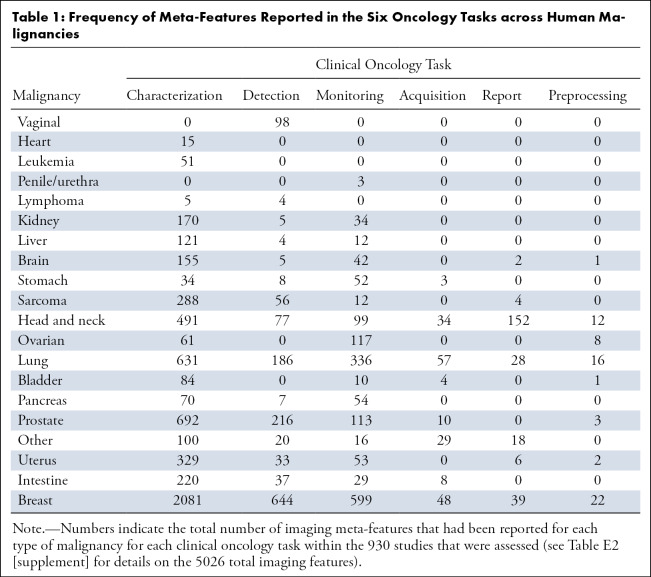
Frequency of Meta-Features Reported in the Six Oncology Tasks across Human Malignancies

**Table 2:**
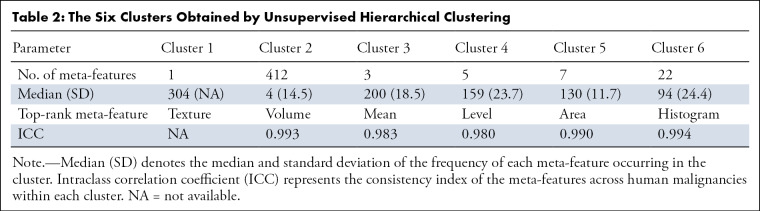
The Six Clusters Obtained by Unsupervised Hierarchical Clustering

### Clustering of Imaging Features by Malignancy Type

According to the clustering of the meta-features, the distribution of imaging features showed a high consistency in breast, lung, and prostate cancer. Pairwise comparison of the three malignancies showed that the Pearson correlation coefficient was greater than 0.75. The imaging features in cluster 3 (Table E4 [supplement]) were reported more frequently than in other clusters and were highly correlated with characterization, detection, monitoring, and reporting tasks in breast, lung, head and neck, and prostate cancer. The features in cluster 4 were significantly focused on tasks of monitoring, acquisition, reporting, and preprocessing in breast and lung cancer. The features in cluster 5 were significantly correlated with the tasks of detection, reporting, and characterization of breast, lung, and prostate cancer. Table E17 (supplement) illustrates the medical imaging meta-features with the highest reporting frequencies (top five) in each oncology task of human malignancy types.

The top five ranked meta-features of task of characterization, detection, acquisition, monitoring, preprocessing, and report are presented in Table 3. Although the distribution of most meta-features was sparse in cancer (cluster 2), particular features were highlighted in specific tasks for malignancies in certain locations. In Figure 2, the meta-feature of “extinction” was most often mentioned in relation to the detection of vaginal cancer, and the meta-feature of “color” was most often used for characterization and detection of sarcoma. The meta-feature of “emphasis” was frequently employed for the reporting task in head and neck cancer. The meta-feature of “distance” was most often applied to the image acquisition task in lung and prostate cancer. The corresponding statistical results in Figure 2 are listed in Table E4 (supplement). For each oncology task (image acquisition, preprocessing, detection, characterization, monitoring, and report), the corresponding details of the application of meta-features for each cancer type are presented separately in Tables E5–E10 (supplement). Appendix E5 (supplement) provides a description of the meta-feature reorganization.

**Table 3:**
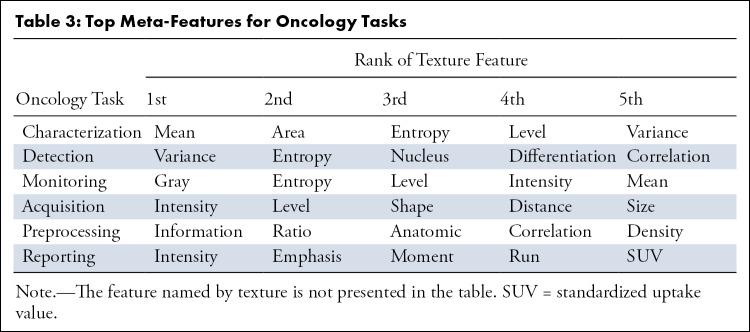
Top Meta-Features for Oncology Tasks

Histologic assessment accounted for 22% (212 of 930) of all included studies. When medical imaging features were extracted from a different imaging modality, the results of our study indicated that the reported features varied across different imaging modalities (Tables E11–E16 [supplement]). The scanners and scanning parameters and other details of image acquisition were vaguely reported in 52% (485 of 930) of the included studies. Appendix E6 (supplement) summarizes the significant meta-features in each imaging modality.

### Validation of NSCLC Texture Features

**Figure 4:**
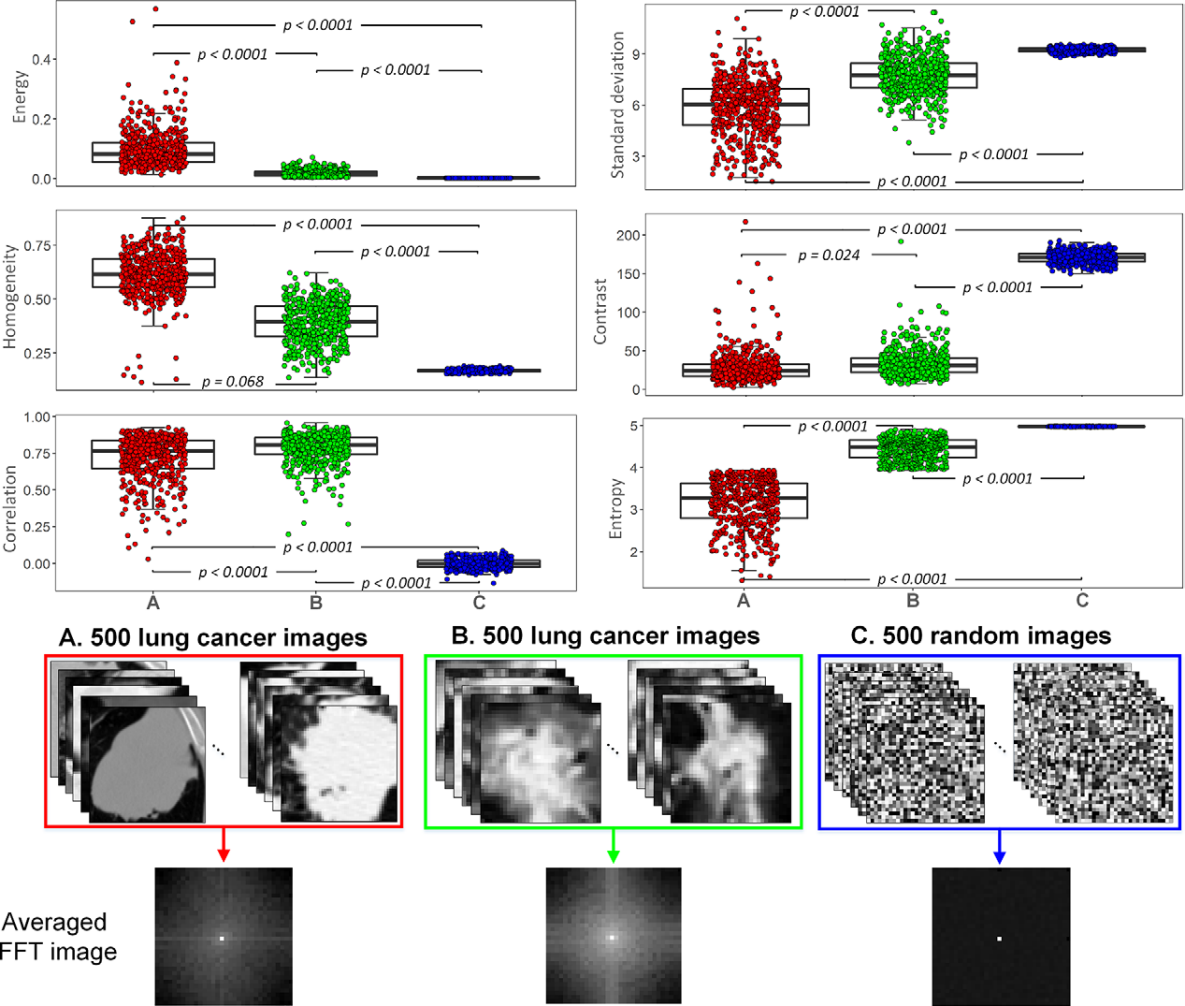
The pattern of the high-dimensional complex texture feature value distributions. The distribution of the values of the six imaging features among the three groups: group A represents CT images from 500 patients with non–small cell lung cancer (NSCLC) with low entropy, group B represents lung cancer images from 500 patients with NSCLC with high entropy (cutoff value: 3.935), and group C represents 500 random images produced by computer program. Contrast, correlation, homogeneity, and energy features were extracted in the 0° direction. The averaged fast Fourier transform (FFT) image of each group (average of the Fourier transforms of all images in the group) is presented for visual distinction of the images with statistically significant different feature values.

Our study indicated that there were certain features with high reporting frequencies across malignancies. Of these, the value of simple features, such as mean, intensity, gray level, and shape descriptors, can be visually assessed by radiologists. However, it is impracticable for the highly reported complex texture descriptors, such as entropy, contrast, correlation, standard deviation (SD), homogeneity, and energy. In this study, pretherapy CT images of 1000 patients with NSCLC and 500 random images generated by computer were used to explore the rule for the distribution of the values of these features. Details of the CT and the random images are presented in Appendix E7 (supplement). All ROIs in CT images were segmented by the two radiologists, and the ICC of the segmentation of the two radiologists ranged from 0.810 to 0.936. We divided the images from the patients with lung cancer into two groups by the median of the entropy values (cutoff value: 3.935). Lung cancer images with low entropy also showed low contrast (*P* = .024), low SD (*P* < .0001), and low correlation (*P* < .0001), but had high homogeneity (*P* = .068) and high energy (*P* < .0001) compared with the other lung cancer group (Fig 4). For the previously mentioned texture features extracted in other directions (45°, 90°, and 135°), the same rule applied (Figs E2–E4 [supplement]). Compared with lung cancer image groups, the value of imaging features of entropy, contrast, and SD of the random image group were highest, whereas the correlation, homogeneity, and energy of this group were the lowest (all *P* < .0001). To display this difference visually, we performed FFT on all images and averaged the FFT images of each group. In this way, it was possible to present the differences among images with significantly different feature values visually.

**Figure 5:**
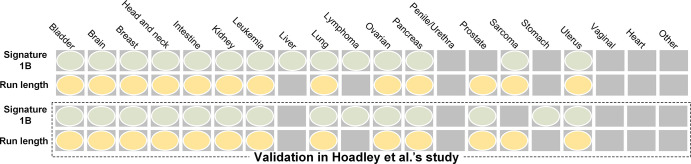
The presence of mutational signature 1B across human malignancy types was highly consistent with the imaging feature of run length. This finding was also verified by Hoadley et al (28).

The ad hoc exploration in this study indicated that the mutational signature 1B, which is characterized by C > T substitutions at NpCpG trinucleotides (27), was most frequent in all human cancers. Comparison of the mutational signatures and the imaging features across human cancer types indicated that the presence of mutational signature 1B was consistent with that of the run length imaging feature (*P* > .5, Fig 5). This rule was further verified in Hoadley et al (28) (*P* > .5, Fig 5).

## Discussion

In this study, we systematically reviewed all the studies published in PubMed for malignancy analysis based on medical imaging features to determine the general rules of applying these imaging features to the analysis of human malignancies. We reviewed the previously reported statistically significant imaging features for all six oncology tasks related to malignancies affecting 20 anatomic regions in humans and summarized the data about these cancer-related imaging features in terms of the malignancy site, imaging modality, and clinical utility. We developed a correlation atlas of human malignancies and imaging features and proposed an approach to investigate the details of any high-dimensional imaging feature of interest. Our study provided a basis for more credible imaging features selection in all oncology tasks across a wide spectrum of human cancers and may help to reduce the bias caused by the investigator’s preference and experience in obtaining potential imaging biomarkers.

In this field of study, a feature selection process is generally required, particularly in the emerging radiomics field (30,31). To retain features of great significance (ie, those that are regarded as potential imaging biomarkers of a predefined clinical hypothesis), extraction of an extensive range of imaging features is generally a prerequisite (32–34). However, most of the relevant studies did not adopt the same criterion to standardize feature extraction. The feature extraction process, especially image filtering and texture matrix calculation, was markedly affected by the different methods used. These flaws may incur serious bias, leading to unrepeatable studies and invalid conclusions (14–16,35–37). Recently, concerns and possible solutions have been proposed to this challenge (13,23,38). To improve validity and soundness of studies within these fields, it is necessary to summarize previous medical imaging data to provide a scientific basis for future feature selection instead of blindly investigating unverified medical imaging features.

The naming of imaging features was inconsistent across analyzed reports within this study (20). To help organize and simplify these data, we provided the data in a format that was easy to query. In addition, the descriptions of the original features were provided, which can help to provide a bridge for statistical analysis of a variety of imaging features. Deconstruction and recombination of the meta-features facilitate an understanding of the details of utilization of any imaging feature in human malignancies. In addition, our results showed that certain imaging features were highly focused on particular oncology tasks in certain malignancies, which is consistent with previous reports (39–41). Researchers can use these specific, highly focused imaging features in future validation studies for translation into clinical practice. Texture features for heterogeneity evaluation are generally difficult to distinguish by the naked eye. For complex texture imaging features that are most frequently reported, the present study revealed a pattern of feature value distributions in different images to clarify the differences in visual characteristics of images with different feature values.

The literature retrieval, feature extraction, data collection, and quality assessment in this study were performed in accordance with the published standards of review (42). Statistical assessment of imaging features from different imaging modalities indicated that the significant features for each human malignancy type differed across imaging modalities. Studies on histopathologic images accounted for 22% (212 of 930) of the total studies included, and the features from these images have been proven to be significantly associated with the diagnosis and prognosis of human malignancies (6,43).

Previous studies have considered that particular changes in tumor phenotypes occur in tumors that express mutation signature 1B (27,44,45). Compared with healthy tissues, gene mutation is reflected in the heterogeneity of the tumor image through intrinsic biologic processes (46–48). The strong consistency between the significant expression of mutational signature 1B across human cancers and the presence of the run length imaging feature across different human cancer types may be particularly useful in image analysis of these cancers. Further molecular-level studies are needed to reveal the biologic function of cancer gene mutations that lead to changes in image phenotypes.

Our study had several limitations. This study may not cover all the literature in this research field because all the journals and conferences related to imaging feature analysis may not be retrieved from the PubMed database. Literature in other databases should also be considered in future analyses. Reliable interobserver consistency ensured the accuracy of the data extracted by investigators in the current study (average ICC = 0.955), but the bias of subjective experience cannot be eliminated. In addition, the meta-feature method can provide details of the application of imaging features in cancer, but the process involves two steps (the recombination of meta-features, and the retrieval of the corresponding original imaging feature), and more concise approaches need to be developed in the future. Finally, because the other gene mutational signatures were not expressed in a wide range of cancers, analysis of the potential correlation of other gene mutational signatures and imaging features requires more specific biologic studies in the future.

In conclusion, we systematically summarized the data of previously reported medical imaging features in human malignancies. Our study clarified the general rules for the utilization of decoded medical imaging features to assist in various radiology tasks for different human malignancies. The results of this study may lay the foundation for improving the reproducibility of experimental results and for reducing bias and redundancy in future medical imaging studies.

## APPENDIX

Appendices E1–E7, Tables E1–E18 (PDF)

## SUPPLEMENTAL FIGURES

Figure E1:

Figure E2:

Figure E3:

Figure E4:

Figure E5:

Figure E6:
