# Supplementary material for: Decoding and Systematization of Medical Imaging Features of Multiple Human Malignancies
Source: Radiol Imaging Cancer. 2020 Sep 11;2(5):e190079. doi: 10.1148/rycan.2020190079 (PMC7983692; doi:10.1148/rycan.2020190079)
Supplement: Appendices E1–E7, Tables E1–E18 (PDF) [file rycan190079suppa1.pdf]

©RSNA, 2020
10.1148/rycan.2020190079

All the supplementary tables are presented as open access data. Please visit the following URL: *https://data.mendeley.com/datasets/gv5j2gk467/10* for the tables presented in Excel spreadsheet. Word versions of all tables are also attached to this library. The table list and corresponding DOI are presented as following.

Appendix E1. Article Inclusion and Exclusion Criteria

For our review, articles were eligible for inclusion if they explicitly proposed solutions for the prespecified clinical task(s) by using imaging feature-based techniques and reported the enrolled subject and image characteristics according to prespecified criteria. Studies were also included in which the medical imaging features were extracted in a quantitative or qualitative manner from regions of interests (ROIs) in radiologic images of malignancies, or from cancer cell images or high-dimensional resolution histopathology images. Studies applying medical imaging features to particular malignancy task(s) usually require enrollment of cases with particular corresponding image scan sequences. Thus, radiology subspecialties or techniques used in the included studies also had to be declared.

Both print and online-only original articles registered in online archives were included in this study. Review articles were also included in this study if medical imaging features applied to specific malignancy tasks were reported. Articles written in other languages were also included. Forms of publication that were excluded from the analysis include case reports, clinical perspectives, state of the art articles, editorials, letters, quizzes, video-audio media, educational material, book reviews, commentaries, and news pieces.

Extracted manuscripts covered 34 malignancies from 20 different anatomic areas. These malignancies included the brain (glioblastoma, glioma, neuroblastoma, and pediatric-type brain tumor), head and neck (esophageal, oropharynx, thyroid, laryngopharyngeal, and nasopharyngeal cancer), breast (breast invasive carcinoma, triple-negative breast cancer, and breast ductal carcinoma in situ), lung (adenocarcinoma, and squamous cell carcinoma), heart, liver (hepatocellular carcinoma), kidney, stomach, intestine (colon, colorectal, rectal, and duodenum cancer), bladder, prostate, uterus (cervical carcinoma, endometrial cancer, and uterine sarcoma), pancreas, penile/urethra, ovarian (epithelial ovarian cancer), vagina, sarcoma (basal cell carcinoma, soft tissue tumors, and melanoma), leukemia, and lymphoma cancers. Others cancer types that did not belong to any of these cancers were classified as “other.”

Appendix E2. Examples of Naming Inconsistencies

The first challenge of medical imaging feature systematization consisted of identifying the names used for features. Although the definition of imaging features has been provided by the IBSI, in actual studies, the naming of features by different researchers is inconsistent. For example, the reported pixel-based features of “percentage” and “probability”; and the features of “maximum value of histogram” and “most frequent voxel”; and the description of “maximum probability” and “maximum co-occurrence matrix element” actually carried the same meaning, although their descriptions varied across articles. On the other hand, features with similar descriptions, such as “information measures of correlation 1” and “information measures of correlation 2”; and features of “contrast” and “local contrast,” referred to different concepts across articles.

Furthermore, complicated imaging features, such as “long run low gray level emphasis (LRLGLE),” can easily to be confused with other features, such as “long run high gray level emphasis” (LRHGLE), and “short run low gray level emphasis” (SRLGLE).

Appendix E3. Meta-Feature Construction Details

For instance, for simple features, such as shape, size, diameter, count, color, etc, the original feature description was retained as a meta-feature. Complicated descriptions, such as the presentation of “mean and standard deviation of the normalized radial length,” were decomposed into the following meta-features: “mean,” “standard,” “deviation,” “norm,” “radial,” and “length.” Fix feature names, such as “angular second moment (ASM),” “angle co-occurrence matrices (ACM),” “apparent diffusion coefficient (ADC),” and “second diagonal moment (SDM),” were treated as independent meta-features. Based on these meta-features, it was then possible to clarify the details of utilization of each imaging feature in human cancer-related tasks.

Appendix E4. Visualization of the Network Topology Diagram

An online computing platform for visualization of the network topology diagram of these correlations is presented at http://www.ciitool.com/#/mifa in this study. In the visualized network, both features and cancers were shown as nodes (see Figure 3 in the manuscript). Once a feature (or cancer node) is clicked, the other nodes with which it is correlated are enhanced in the display, to facilitate understanding. For a specific node of interest, scrolling of the mouse enables the viewer to read more details. Besides, we separately present the details describing the applications of meta-features across all cancer types, sorted by radiologic image (CT, mammography, MRI, PET, and ultrasound) and histopathology image, in Tables E11–E14. An open access database expressing the correlation of the human cancer type, oncology tasks, and corresponding medical imaging features is available at the URL: http://dx.doi.org/10.17632/gv5j2gk467.10.

Appendix E5. Recombining Meta-Features to Obtain Statistics of Original Imaging Features

The names of the original features in this study were manually extracted from the existing literature reports, and were not artificially modified to maintain accuracy. Thus, most meta-features were single features, as they were extracted from complex feature representations. Therefore, it may be difficult for readers to decide the relationship between individual meta-features and human cancers. However, based on the statistics and meta-features, if the reader is interested in a certain imaging feature that has complicated descriptions, the details of utilization of the imaging feature in human cancers could be restored. By using the statistical results provided in this study, the reader can review the specific details of any of the imaging features of interest in cancer. Then, using the records of the original feature in Table E2, the PubMed ID of the studies that reported the original imaging feature, as well as the name of other features reported in the same article, and the corresponding clinical tasks and imaging modalities, can be obtained. For instance, for the complicated feature of “short run emphasis,” which is a high-dimensional texture feature from the run-length matrix, by recombining the meta-features of “short,” “run,” and “emphasis,” we found that this feature was mainly used for the clinical task of characterization of lung cancer by using CT images in Table E11. Then, the corresponding

PubMed ID, the clinical tasks, the other imaging features reported in the same article, and the imaging modalities of the “short run emphasis” could be traced by searching the meta-features or original features in Table E2.

Appendix E6. The Significant Meta-Features in Imaging Modality

The mean, entropy, intensity, gray level, and shape were the most frequently reported meta-features in studies using CT scans, which significantly concentrated on characterization and monitoring tasks in lung cancer. In MRI, enhancement, contrast, intensity, entropy, variance, and correlation were the top features, which were mostly used for the characterization task in breast and prostate cancer. Nucleus, area, density, mean, variance, and shape were the most commonly used meta-features of histopathology images, which were mainly used for characterization and detection tasks in breast and vaginal cancer.

Appendix E7. Supplementary Materials of CT Image

CT Imaging for Visualization of the Significant Texture Features

Pretherapy contrast-enhanced CT images of 1000 early-stage patients with NSCLC were acquired in the Department of Radiology at our hospital. Contrast-enhanced CT were performed on every patient using one of the two multidetector row CT (MDCT) systems (GE Lightspeed Ultra 8, GE Healthcare, Hino, Japan or 64-slice LightSpeed VCT, GE Medical systems, Milwaukee, Wis), with the following acquisition parameters: 120 kV; 160 mAs; 0.5-or 0.4-second rotation time; detector collimation: 8 × 2.5 mm or 64 × 0.625 mm; field of view, 350 × 350 mm; matrix, 512 × 512. After routine nonenhanced CT, contrast-enhanced CT was performed after 25 s delay following intravenous administration of 85 mL of iodinated contrast material (Ultravist 370, Bayer Schering Pharma, Berlin, Germany) at a rate of 2.5–3.0 mL/s with a pump injector (Ulrich CT Plus 150, Ulrich Medical, Ulm, Germany). CT image was reconstructed with standard kernel, with interval: 1 mm-2.5 mm. Retrieval of CT images: All of the CT images were retrieved from the picture archiving and communication system (PACS) (Carestream, Canada).

Tumor Boundary Extraction and Feature Analysis

Tumor delineation.—

Complete region of interest (ROI) of lung tumor was manually delineated for texture feature analysis. Two radiologists with more than 10 years of experience in chest CT interpretation were chiefly responsible for the manual tumor segmentation. We randomly selected 20 cases and the similarity index of features from the segmentation results by the two radiologists indicated that the interclass correlation coefficient (ICC) of the segmentation of the two radiologists was ranged from 0.810 to 0.936. The average stability of extracted features was 92.50%.

Feature analysis.—

The texture features of the image in the middle layer of each patient were first calculated by the algorithm in Matlab (version: 2015b), and then the values of the texture features of different patients were displayed by using R language (version: 3.4.3).

The following are the details of feature analysis, and all the source code was published in following URL: DOI: http://dx.doi.org/10.17632/gv5j2gk467.9.

A. Matlab (version: 2015b) was used to calculate the six texture features for all the images. First, we randomly generate 500 images with the dimension of 32*32 by computer program. Then, the feature of entropy of the random images and NSCLC images were calculated by the “entropyrandom” and “entropy” function, respectively. The core-algorithm of the calculation of the feature of entropy of was the toolkit of “entropy” in Matlab. Next, the toolkit of “graycomatrix” was used to obtain the texture feature of standard deviation, correlation, contrast, energy, and homogeneity in four directions. In this study, the gray level value of random images and that of lung cancer ROIs were normalized uniformly. The algorithm is as follows.

```
%% random images
resultofrandom = zeros (500,20);
for i = 1:500
RandomImage1 = ceil (rand (32,32)*32); %%random image initialization.
resultofrandom (i,2) = entropyrandom (RandomImage1); %% entropy calculation of the
random images.
resultofrandom (i,1) = i;
resultofrandom (i,3) = 1024;
RandomImage1 = fix (RandomImage1);
%%graycomatrix was used to obtain the feature of standard deviation, correlation, contrast,
energy, and homogeneity features in four directions.
[GLCMS,SI] = graycomatrix (RandomImage1,'GrayLimits',[1,32],'NumLevels',32,'Offset',[0
1;-1 1;-1 0;-1–1]);
status = graycoprops (GLCMS);
resultofrandom (i,4:7) = deal (status.Contrast);
resultofrandom (i,8:11) = deal (status.Correlation);
resultofrandom (i,12:15) = deal (status.Energy);
resultofrandom (i,16:19) = deal (status.Homogeneity);
resultofrandom (i,20)=std (RandomImage1 (1:end),0);
end
%%CT images of the 1000 lung cancer patients.
resultofrealimage = zeros (1025,20);
for i = 1:1000
DicomReadSegtrace = ['Path of the data set in your personal computer'];
PatientFile = dir (DicomReadSegtrace);
if (length (PatientFile) > 2)
```

```
DicomReadSegtrace = ['Path of the data set in your personal computer + the segmented mask images'];

PatientFile = dir (DicomReadSegtrace);

DicomReadSeg = dicomread (DicomReadSegtrace);

DicomReadOriTrace = ['Path of the data set in your personal computer + the original images'];

PatientFile = dir (DicomReadOriTrace);

DicomReadOri = dicomread (DicomReadOriTrace);

resultofrealimage (i,1) = i;

[resultofrealimage (i,2),resultofrealimage (i,3)] = entropy (DicomReadOri,DicomReadSeg);%%entropy of the lung cancer images.

DicomReadOri = double (DicomReadOri);

DicomReadOri (DicomReadSeg ~ =4096)=-1000;

image_minGray = min (min (DicomReadOri (DicomReadOri ~ =-1000)));

image_maxGray = max (max (DicomReadOri (DicomReadOri ~ =-1000)));

image_distance = image_maxGray-image_minGray;

DicomReadOri (DicomReadOri ~ =-1000) = (DicomReadOri (DicomReadOri ~ =-1000)-image_minGray)/image_distance;

min_Gray = 1;

max_Gray = 32;

DicomReadOri (DicomReadOri ~ =-1000) = max_Gray*DicomReadOri (DicomReadOri ~ =-1000) +min_Gray;

DicomReadOri = fix (DicomReadOri);

%%graycomatrix was used to obtain the feature of standard deviation, correlation, contrast, energy, and homogeneity features in four directions.

[GLCMS,SI] = graycomatrix (DicomReadOri,'GrayLimits',[1,32],'NumLevels',32,'Offset',[0 1;-1 1;-1 0;-1–1]);

GLCMS (1,1)=0;

status = graycoprops (GLCMS);

resultofrealimage (i,4:7) = deal (status.Contrast);

resultofrealimage (i,8:11) = deal (status.Correlation);

resultofrealimage (i,12:15) = deal (status.Energy);

resultofrealimage (i,16:19) = deal (status.Homogeneity);

resultofrealimage (i,20)=std (DicomReadOri (DicomReadOri ~ =-1000),0);

end
```

```
end
```

B. After obtaining all the texture feature values, the toolkit of “ggplot2” in R language (version: 3.4.3) was used to display the feature values of different images. The algorithm is as follows.

```
library (ggplot2)
library (RColorBrewer)
workbook1 <-”load the 'resultofrealimage' and the 'resultofrandom' data which have been obtained by the 'Improtant_features_calculation.m'”
Sta1 = read_excel (workbook1,1)
FeatureValues <-Sta1
FeatureValues1 = data.frame (Group = FeatureValues$Class, Value = FeatureValues$Contrast4)
FeatureValues1$Group = factor (FeatureValues1$Group,levels = c (1,2,3))
P1 <-ggplot (FeatureValues1,aes (x = Group,y = Value,fill = Group))+
stat_boxplot (geom = “errorbar,”width = 0.15,aes (color = ”black”))+
geom_boxplot (size = 0.9,fill = ”white,”outlier.fill = ”white,”outlier.color = ”white”)+
geom_jitter (aes (fill = Group),width = 0.2,shape = 21,size = 2.0)+
scale_fill_manual (values = c (“#FF0000,” “#00FF00,””#0000FF”))+
scale_color_manual (values = c (“black,””black,””black”))+
ggtitle (“”)+
theme_bw ()+
theme (legend.position = ”none,”
axis.text.×=element_text (color = ”black,”family = ”Times,”size = 14),
axis.text.y = element_text (family = ”Times,”size = 14,face = ”plain”),
axis.title.y = element_text (family = ”Times,”size = 14,face = ”plain”),
axis.title.×=element_text (family = ”Times,”size = 14,face = ”plain”),
plot.title = element_text (family = ”Times,”size = 15,face = ”bold,”hjust = 0.5),
panel.grid.major = element_blank (),
panel.grid.minor = element_blank ())+
ylab (“Contrast”)+xlab (“”)
P1
tiff (file = “save your boxplot,”width = 2400,height = 1000,units = “px,”res = 300)
print (P1)
dev.off ()
```

```
x1 <-FeatureValues1[which (FeatureValues1[,"Group"]=="1"),"Value"] ##1. The 500 patient images with low entropy
x2 <-FeatureValues1[which (FeatureValues1[,"Group"]=="2"),"Value"] ##2. The 500 patient images with high entropy
x3 <-FeatureValues1[which (FeatureValues1[,"Group"]=="3"),"Value"] ##3. The 500 random images.
var.test (x1,x2)
var.test (x1,x3)
var.test (x2,x3)
x <-aov (Value ~ Group,data = FeatureValues1)
summary (x)
```

Table E1. Table_L01_List of Meta-features

DOI: http://dx.doi.org/10.17632/gv5j2gk467.10#file-ec7e1fcc-1c83-4234-87ca-1c081871e479

Table E2. Table_F01_5026 original features sorted by articles

DOI: http://dx.doi.org/10.17632/gv5j2gk467.10#file-3c8f3f38-6e21-419d-9a54-f5fc3ee3cebd

Table E3. Table_S01_correlation atlas sorted by meta-feature

DOI: http://dx.doi.org/10.17632/gv5j2gk467.10#file-9c8ee8f3-f83b-4904-859b-5429ccae8d12

Table E4. Table_S02_correlation atlas of malignancies and meta-features in all of the six oncology tasks

DOI: http://dx.doi.org/10.17632/gv5j2gk467.10#file-fd65da5c-6cd1-428e-8948-796963da36ae

Table E5. Table_S03_correlation atlas of malignancies and meta-features in characterization task

DOI: http://dx.doi.org/10.17632/gv5j2gk467.10#file-614b61c2-db47-4cf7-af35-50c70d7d5027

Table E6. Table_S04_correlation atlas of malignancies and meta-features in detection task

DOI: http://dx.doi.org/10.17632/gv5j2gk467.10#file-efbcc338-5038-42c1-8c32-e4f07d597ed8

Table E7. Table_S05_correlation atlas of malignancies and meta-features in acquisition task

DOI: http://dx.doi.org/10.17632/gv5j2gk467.10#file-ec0b91d3-da5c-4d86-9e35-513d6111ca25

Table E8. Table_S06_correlation atlas of malignancies and meta-features in monitoring task

DOI: http://dx.doi.org/10.17632/gv5j2gk467.10#file-a0034425-9c13-4f52-bbb7-7dce28577591

Table E9. Table_S07_correlation atlas of malignancies and meta-features in preprocessing task

DOI: http://dx.doi.org/10.17632/gv5j2gk467.10#file-de1f0af5-bc83-48aa-8649-e78a8c806019

Table E10. Table_S08_correlation atlas of malignancies and meta-features in report task

DOI: http://dx.doi.org/10.17632/gv5j2gk467.10#file-498aa3ef-3325-4a2a-a6e8-63d2c23d0635

Table E11. Table_S09_CT: correlation atlas of malignancies and meta-features

DOI: http://dx.doi.org/10.17632/gv5j2gk467.10#file-de8f278a-7cfa-4075-8c7f-055a2cd5d1f7

Table E12. Table_S10_Histopathology: correlation atlas of malignancies and meta-features

DOI: http://dx.doi.org/10.17632/gv5j2gk467.10#file-53e072f1-2ef1-4a0e-9616-3558e9191fbb

Table E13. Table_S11_Mammography: correlation atlas of malignancies and meta-features

DOI: http://dx.doi.org/10.17632/gv5j2gk467.10#file-867f1d3a-a3d8-4910-be72-8545e1d582e2

Table E14. Table_S12_MRI: correlation atlas of malignancies and meta-features

DOI: http://dx.doi.org/10.17632/gv5j2gk467.10#file-b901652b-17f7-4795-aa65-1c5567cce632

Table E15. Table_S13_PET: correlation atlas of malignancies and meta-features

DOI: http://dx.doi.org/10.17632/gv5j2gk467.10#file-436aade9-6a52-4f99-b530-4b91d328e94d

Table E16. Table_S14_Ultrasound: correlation atlas of malignancies and meta-features

DOI: http://dx.doi.org/10.17632/gv5j2gk467.10#file-e7b8a288-c834-44df-8318-0c0ae955ba77

Table E17. Table_T1_Top rank meta-features of each malignancy type

DOI: http://dx.doi.org/10.17632/gv5j2gk467.11#file-f1e139f0-0995-461b-8449-89ba63030d2f

Table E18. Demographic information of the 1000 patients with NSCLC in this study.

**Characteristics**	**NSCLC**
**Age average (SD)**	54 (10.3)
**Gender**	
Male	653
Female	347
**Smoking**	
Yes	688
No	312
**Clinical stage**	
I	651
II	449

Note.—The pretherapy contrast-enhanced CT images of the patients with NSCLC were collected for feature visualization analysis in this study. The patients were eligible when meet the following inclusion criteria: *(a)* clinical diagnosed of early stage non-small cell lung cancer from January 1st, 2013 to December 31st, 2018, *(b)* enhanced CT scan was performed at our facility two weeks before receiving treatment, *(c)* patient's demographics was available. Finally, we selected 1,000 patients who met the above criteria, and the CT images were used for the calculation and display image features.
