# Supplementary figures and images for: Decoding and Systematization of Medical Imaging Features of Multiple Human Malignancies
Source: Radiol Imaging Cancer. 2020 Sep 11;2(5):e190079. doi: 10.1148/rycan.2020190079 (PMC7983692; doi:10.1148/rycan.2020190079)

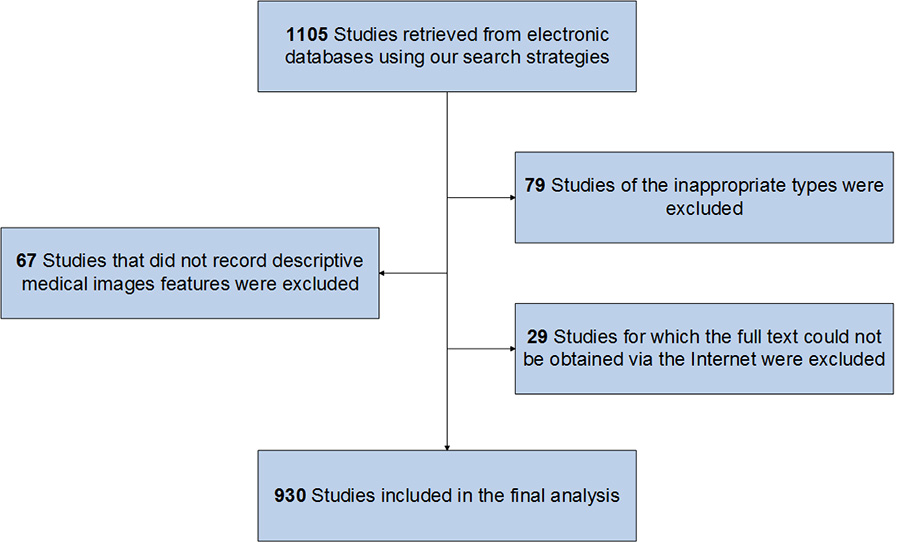

Supplement: Figure E1: [file rycan190079suppf1.jpg]

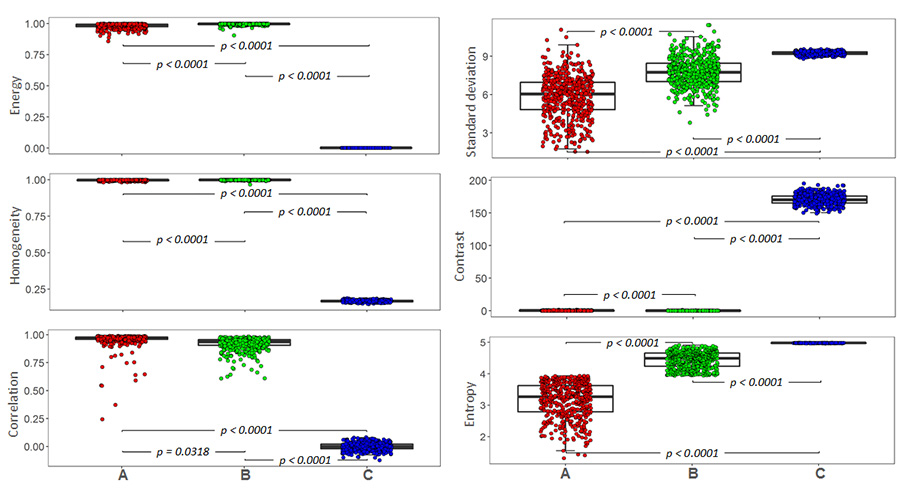

Supplement: Figure E2: [file rycan190079suppf2.jpg]

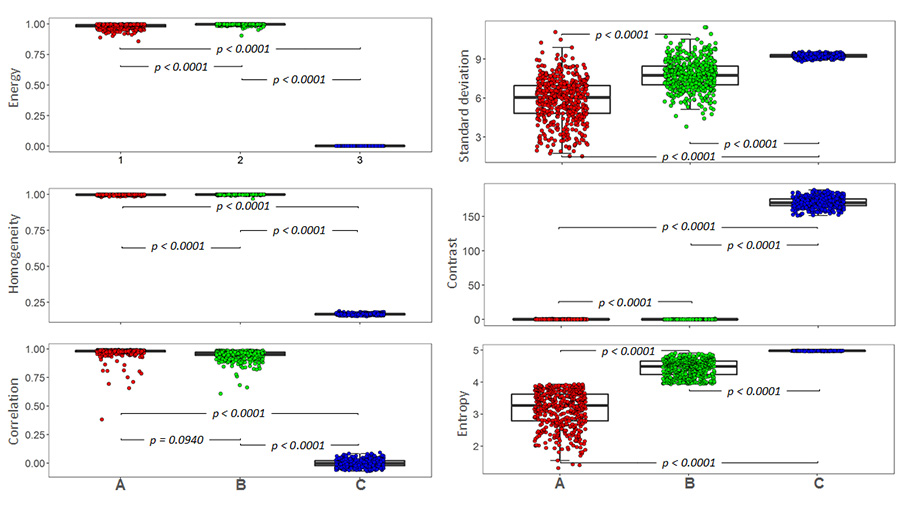

Supplement: Figure E3: [file rycan190079suppf3.jpg]

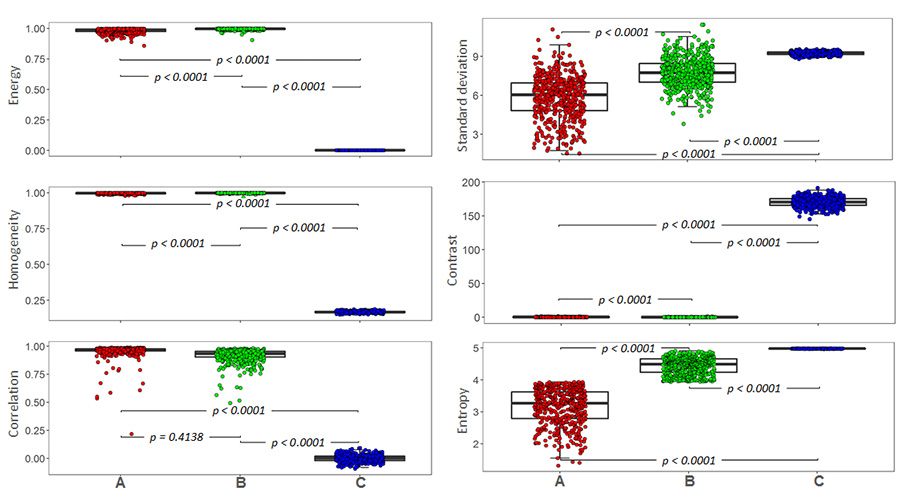

Supplement: Figure E4: [file rycan190079suppf4.jpg]

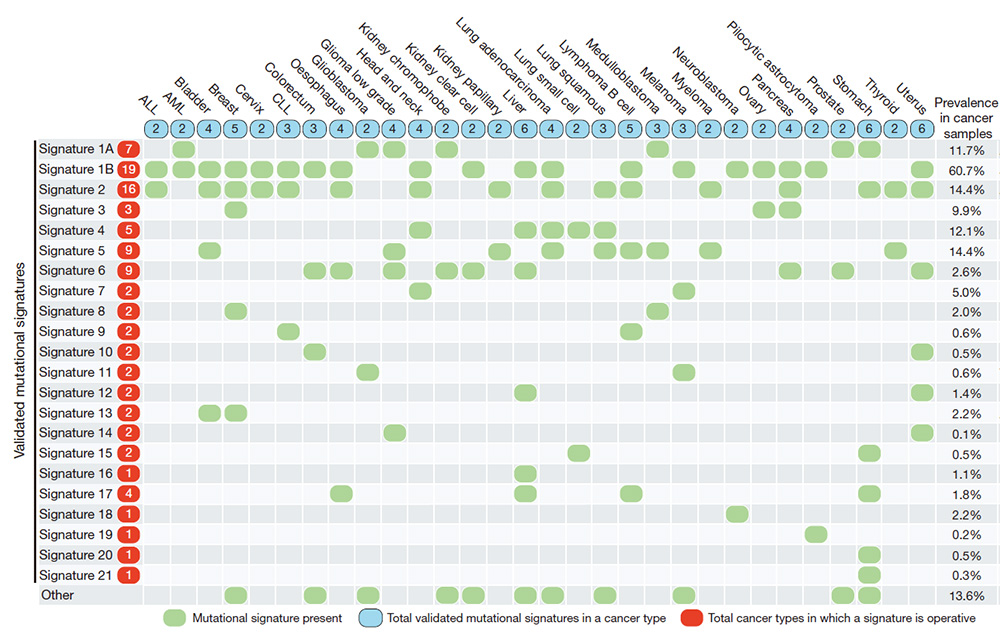

Supplement: Figure E5: [file rycan190079suppf5.jpg]

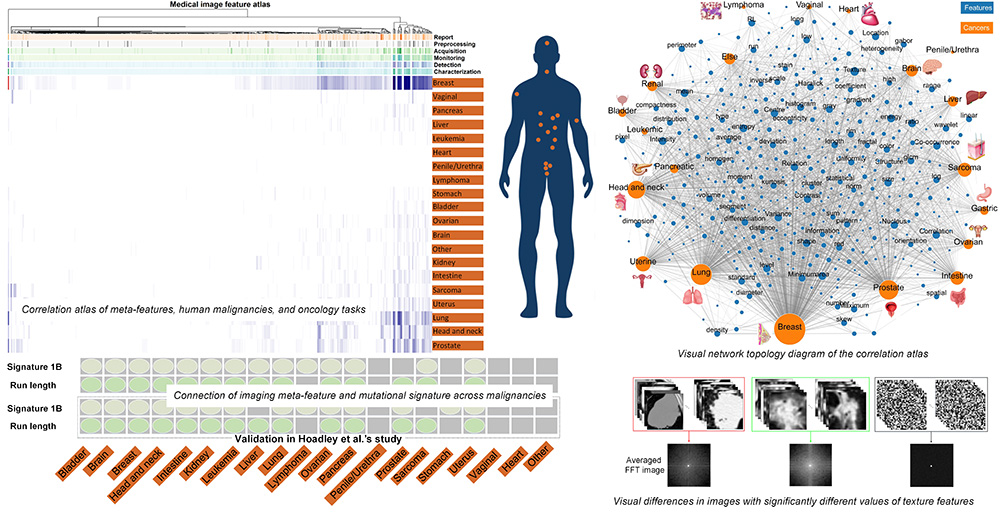

Supplement: Figure E6: [file rycan190079suppf6.jpg]
